# The Role of Prenatal Ultrasound Examination in Predicting the Outcomes of Ovarian Fetal Cysts: A Pictorial Essay

**DOI:** 10.3390/diagnostics14232726

**Published:** 2024-12-04

**Authors:** Elisa Montaguti, Benedetta Petrachi, Marta Fiorentini, Viola Arosio, Sara Doroldi, Camilla Dionisi, Vito Bernardi, Gianluigi Pilu

**Affiliations:** Obstetric Unit, IRCCS Azienda Ospedaliero-Universitaria di Bologna, Via Massarenti 13, 40138 Bologna, Italy; benedetta.petrachi@gmail.com (B.P.); viola.arosio@gmail.com (V.A.); sara.doroldi@gmail.com (S.D.); camilladionisi@gmail.com (C.D.); vito.bernardi91@gmail.com (V.B.); gianluigi.pilu@aosp.bo.it (G.P.)

**Keywords:** ovarian fetal cysts, ultrasound, postnatal outcomes, postnatal surgery

## Abstract

**Objectives:** The aim of this study was to evaluate prenatal ultrasound features, fetal postnatal outcomes, and the need for postnatal surgery in the suspicion of ovarian torsion. **Methods:** We included patients with a singleton pregnancy with a suspicion of ovarian fetal cyst referred to our center. Data derived from prenatal ultrasound evaluations, delivery, and postnatal follow-up were then extracted from the hospital registers. **Results:** The ultrasound features of 32 fetal ovarian cysts and related neonatal outcomes were analyzed. The mean gestational age at diagnosis was 32 weeks (28–36), while the mean diameter of the cyst diagnosis was 34.8 ± 13.2 mm. In 78.1% of cases, the cysts did not change their characteristics during pregnancy, while in 9.4%, they increased their dimensions, and in 12.5%, they reduced their size. In 78.1% of cases, the diagnosis was confirmed postnatally, and in 40% of cases, a spontaneous regression occurred during follow-up. Surgery was performed in 60% of cases, and most of the time (66.7%), an adnexectomy was required. **Conclusions:** An unfavorable outcome was associated with cystic dimensions and ultrasound feature modifications during pregnancy. However, our study demonstrated that a hemorrhagic content is not always indicative of adnexal torsion, and spontaneous resolution may occur. In addition, only a few of the simple anechoic cysts managed surgically presented with necrosis at histopathological examination; therefore, a conservative approach might be proposed in those cases.

## 1. Introduction

The detection of abdominal cysts in the fetus is a quite frequent finding during routine prenatal ultrasound evaluations, in particular during the late second and third trimesters. The possible differential diagnoses are heterogeneous and include ovarian cysts (in female fetuses), common bile duct, hepatic, splenic, pancreatic, and mesenteric cysts, meconial pseudocysts, hemorrhagic adrenal cysts, renal cysts, and bowel duplication cysts [1,2,3,4].

Choledochal cysts are rare congenital cystic dilations of the biliary tract, classified according to the segment of the biliary tree involved [5,6,7]. Differential diagnosis can be complex, yet Power Doppler ultrasound may assist by revealing the connection of the cyst with the hepatic artery and portal vein, supporting a biliary origin. Hepatic cysts typically present as isolated simple cysts that arise from aberrant bile ducts or peribiliary intrahepatic glands [7,8]. Many of the prenatally diagnosed hepatic cysts decrease or resolve spontaneously after birth, though in rare cases they may enlarge and become symptomatic. Splenic cysts are generally simple serous cysts lined with epithelium and located at the spleen’s upper pole [9]. They are often incidental findings with limited clinical significance. These cysts are commonly diagnosed in the third trimester of pregnancy and may be difficult to distinguish from adrenal or pancreatic cysts. Pancreatic cysts arise from aberrant development of the pancreatic ductal system, typically in the pancreatic body or tail [10]. These cysts have an epithelial lining within a true wall. Intestinal duplication cysts are generally isolated cystic structures with thick walls [11,12]. These cysts can occur at any point in the gastrointestinal tract, most frequently in the jejunum and ileum (53%), but also in the colon (18%), duodenum (6%), and stomach (4%). Enteric duplication cysts can exhibit signs of bowel obstruction with proximal bowel loop dilation. Mesenteric cysts are cystic lymphangiomas (lymphatic malformations) that appear as unilocular or multilocular lesions arising within the intestinal mesentery [13,14]. Meconium pseudocysts are a manifestation of meconium peritonitis, which results from fetal intestinal perforation (typically of the small intestine or colon), allowing intestinal contents to extravasate into an inflammatory cystic space [15]. Additional cystic lesions that may be considered in the differential diagnosis of ovarian cysts include renal cystic lesions, especially in the context of cystic dysplasia [16], in which the kidney appears enlarged, with most of the parenchyma replaced by multiple, non-communicating cysts of variable size and number, with the residual renal parenchyma appearing hyperechoic. In unilateral cases, amniotic fluid levels are typically normal, and the bladder is visualized. Bilateral cases are associated with severe oligohydramnios and the inability to visualize the bladder. These cases should be distinguished from hydronephrosis, defined as renal pelvis dilation that may or may not be accompanied by ureteral dilation. The primary causes include vesicoureteral junction obstruction, vesicoureteral reflux, secondary obstruction from a ureterocele, and ectopic ureter.

Establishing a definitive diagnosis of a prenatally detected intra-abdominal cyst can be challenging [3]. However, in female fetuses, the most common abdominal cysts are those of ovarian origin (1 in 2600 pregnancies).

They are usually diagnosed in the third trimester after 28 weeks of gestation [17], as the hypothalamus–hypophysis–ovary axis activates and are typically benign and functional. Their formation can be explained by a stimulation of fetal ovaries by maternal estrogens, fetal gonadotropins, and human chorionic gonadotropin [17,18]. Cysts can reach 10 cm of diameter [17,19,20] and in 5% of cases, they are bilateral. Ovarian cysts are usually located laterally in the lower abdomen, and a very specific sign to ascertain the ovarian origin of the cysts is the “daughter cysts sign”, which consists in detecting a smaller cyst inside the main one [20,21].

Depending on ultrasound characteristics, ovarian cysts were classically distinguished into simple and complex. Simple cysts are anechoic, with thin walls, regular margins, and no vascularization at Doppler evaluation. Complex cysts, however, usually present with thicker walls and mixed internal echogenicity, with hyperechogenic portions and trabeculae that are usually considered signs of intra-cystic hemorrhage associated with a higher incidence of prenatal ovarian torsion [17].

Intrauterine cystic aspiration under ultrasound guidance has been proposed as a treatment option for large cysts; nevertheless, it is still not clear whether this procedure may actually improve neonatal outcomes [17,22,23,24]. Postnatal management of ovarian cysts has not been standardized either, and although the prognosis is usually good, the chance of ovariectomy is quite high; the indications for surgery differ depending on the center [24,25] and are not always related with the prenatal aspect. In 2000, the International Ovarian Tumor Analysis (IOTA) developed standardized terminology to describe adnexal masses detected at ultrasound evaluation in adults [26]. Some authors recently proposed this classification also for fetal formations and related this characteristic to the chance of postnatal surgery [27]. They demonstrated that cysts with ground-glass, hemorrhagic, or mixed content were more likely to be associated with necrosis at histologic post-surgery examination. Unlike the simple/complex distinction, this classification may allow a better identification of those newborns who may need surgery for ovarian torsion and with confirmed necrosis at histological examination.

The aim of our study was to evaluate our center experience regarding the prenatal ultrasound features of suspected ovarian fetal cysts, their postnatal outcomes, and the need for postnatal surgery.

## 2. Materials and Methods

This was an observational, spontaneous, retrospective, monocentric, and non-pharmacological study. We included patients with singleton pregnancy who were referred to our unit of Obstetrics and Prenatal Medicine for an abdominal cyst suspicion of ovarian origin between 2008 and 2021.

For each patient, we extracted anonymously from the hospital registers data derived from prenatal ultrasound evaluations, delivery, and postnatal follow-up. In particular, we evaluated gestational age at diagnosis, prenatal cystic size and ultrasound features, modification in cystic size or ultrasound features during pregnancy, gestational age at delivery, mode of delivery, birth weight, postnatal diagnosis confirmation and diagnostic approach, postnatal cystic dimensions, cystic spontaneous regression and time required, need for surgery, type of surgery (laparoscopy vs. laparotomy, adnexectomy vs. cystectomy), and histological diagnosis. The ultrasound features were assessed according to IOTA terminology [27] by an experienced operator who attended the IOTA certification program.

We evaluated the aspect of the internal walls of the cyst, the presence of septa or solid components, vascularization at Color Doppler examination, and characteristics of the cystic content (anechoic, low level, ground glass, hemorrhagic, and mixed). According to ultrasound features, ovarian cysts can be therefore classified as unilocular, unilocular-solid, multilocular, multilocular-solid, solid, or unclassifiable.

### Statistics

Clinical and ultrasound data were analyzed with the statistical software Stata (version 17.0). Concerning descriptive statistics, we used mean and standard deviation, median with minimum and maximum values, and frequency with percentages if appropriate. A Student *t* test with a level of significance of 5% was used to evaluate the association between cystic size and need for surgery.

## 3. Results

We analyzed the ultrasound features of 32 fetal ovarian cysts and related neonatal outcomes. The mean gestational age at diagnosis was 32 ± 2 weeks (range 28–36 weeks of gestation). The mean diameter of the cysts at the time of the diagnosis was 34.8 ± 13.2 mm (range 12.7–61.3 mm). According to the traditional classification of fetal ovarian cysts, 22 of them were simple cysts (68.8%), while 10 were complex (31.2%).

Concerning the size, 25 cysts did not change over the course of pregnancy (78.1%); 3 cysts increased their dimensions (9.4%), whereas 4 cysts reduced their size during prenatal follow-up (12.5%). Two of the 32 cysts (6.25%) showed modifications in their ultrasound characteristics. Postnatally, the diagnosis was confirmed in 25 cases (78.1%). Three cases were lost to follow-up as patients delivered elsewhere (9.4%), while in 4 cases, the diagnosis was that of multifollicular ovaries without a cystic formation (12.5%). Transabdominal ultrasound confirmed the diagnosis in 21 cases (84%), whereas 4 cases required the use of abdominal RM (16%). Spontaneous regression during postnatal follow-up was observed in 10 cases (40%), with a mean regression time of 4 ± 3 months (1–11 months). Surgery was performed in 60% of the cases (15/25). All surgical interventions were performed laparoscopically. In 10 cases, an adnexectomy was required (66.7%), while only in 5 cases, a cystectomy was possible (33.3%). The histological report confirmed the diagnosis of follicular ovarian cyst in 14 out of 15 cases (93.3%). Just in one case, the final diagnosis was that of an enteric duplication cyst.

**Figure 1:**
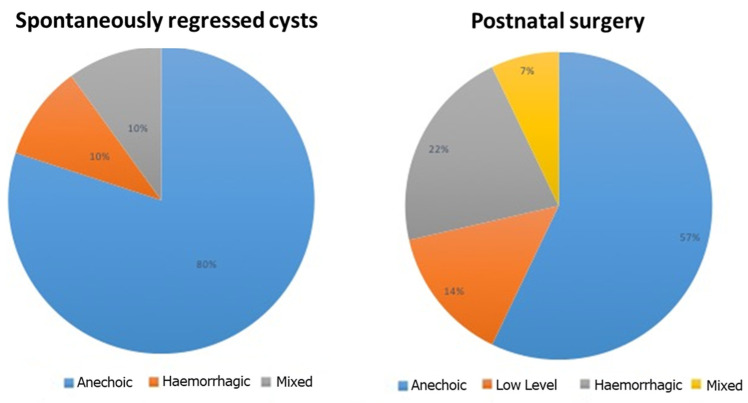
Graphic representations of our study population.

We applied the IOTA criteria and terminology to describe cystic characteristics (Table 1 and Figure 1); the ultrasound images were analyzed independently by two operators with IOTA certification of competence, with perfect agreement between the two. All the 32 fetal cysts we analyzed showed regular borders (100%). Only 4 of them were multilocular (12.5%), while 28 of them were unilocular (87.5%). In our sample, there were no unilocular-solid, multilocular-solid, solid, or unclassifiable cysts. As they showed no vascularization, they were all characterized as Color Score 1 (100%). When evaluating those 10 cysts that showed a spontaneous resolution, their mean prenatal diameter was 29.9 ± 12.6 mm (12.7–45.3 mm); 9 were unilocular cysts (90%), and only 1 cyst was multilocular (10%). In most cases, their content was anechoic (8/10, 80%); in 1 case, it was hemorrhagic (10%); and in only 1 cyst, mixed content (10%) was presented. Among the 10 cysts that showed complete postnatal regression, 20% of them had already diminished their size during pregnancy.

In our study population, the formations that underwent postnatal surgery and were confirmed as ovarian cysts at histological examination were 14. The mean prenatal diameter of these cysts was 40.8 ± 11.5 mm (20.3–61.3 mm). Here, 12 of them were unilocular (85.7%) and 2 were multilocular (14.3%). The content was anechoic in 57.1% of cases (8/14), low level in 14.3% of cases (2/14), hemorrhagic in 21.4% of cases, and mixed in 7.2% of cases (1/14). Three of these cysts increased their size during pregnancy, and 2 of them changed their ultrasound characteristics from simple cysts to complex cysts (as if an in-utero torsion or hemorrhage had happened). Only one cyst showed a dimensional reduction.

**Figure 2:**
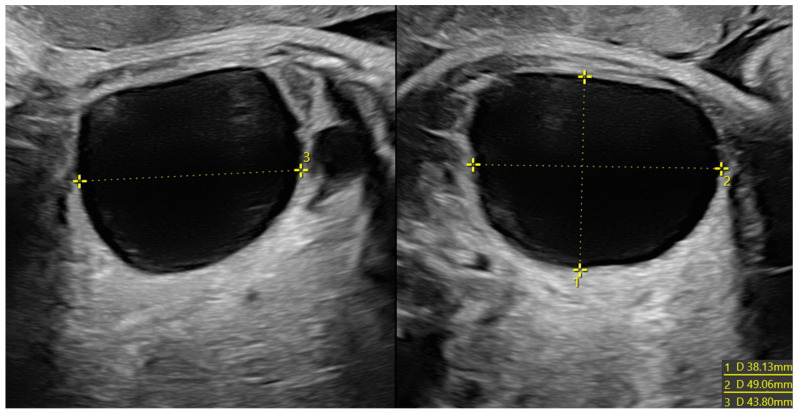
Voluminous ovarian cyst in a fetus at 34 weeks of gestation with anechoic content (38 × 49 × 44 mm in diameter) and no vascularization at Power Doppler. The formation was confirmed and did not regress after birth. Because of this, a cystectomy was performed, with no evidence of necrosis at the histological examination.

**Figure 3:**
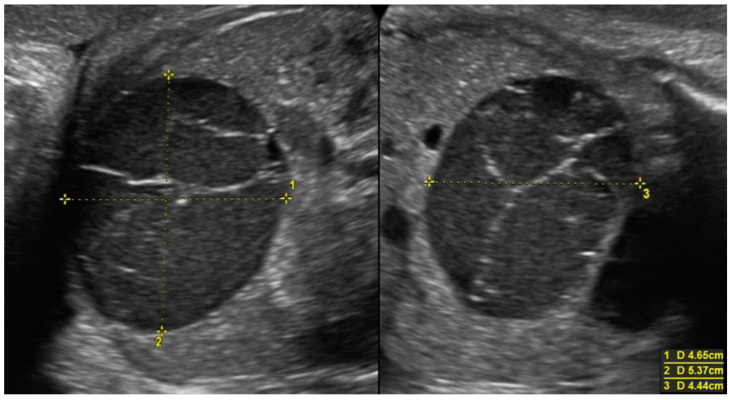
Voluminous ovarian cyst with hemorrhagic content in a female fetus at 35 weeks of pregnancy (46 × 54 × 44 mm). At 37 weeks of pregnancy, the formation regressed spontaneously, and at the ultrasound after delivery, multifollicular ovaries were described.

**Figure 4:**
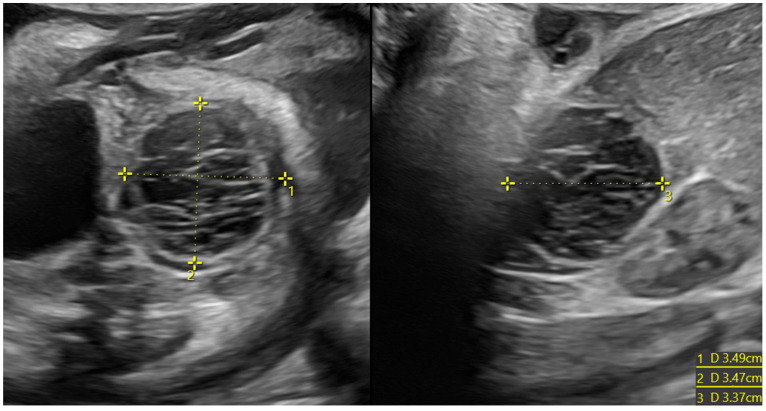
Ovarian cyst with hemorrhagic content in a female fetus at 33 weeks of pregnancy (3.5 cm of diameter). The formation did not regress, and a laparoscopic cystectomy was performed; at the histological examination, necrosis was reported.

**Figure 5:**
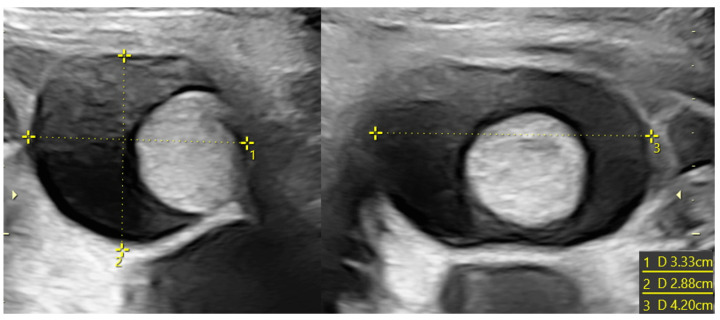
Fetal ovarian cyst at 35 weeks of gestation described as mixed in its content (42 × 33 × 29 mm). The central rounded formation, presumably a blood clot, was not vascularized at Power Doppler, and at postnatal follow-up evaluations, the cyst progressively decreased until disappearing within a month.

**Figure 6:**
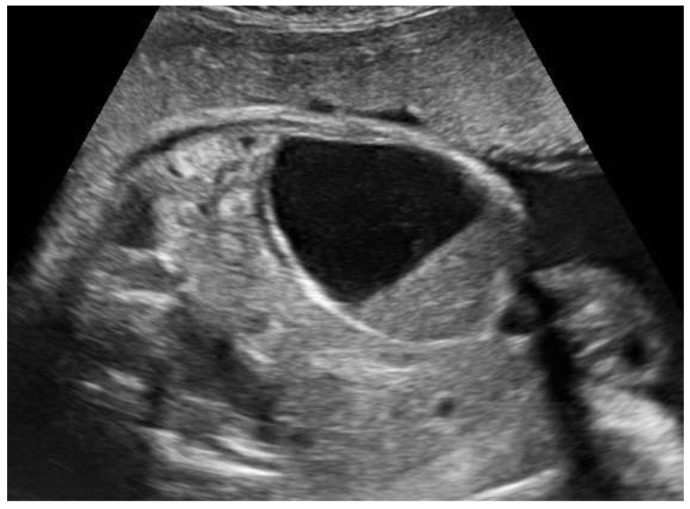
Fetal ovarian cyst at 36 weeks of gestation with a low-level content (4 cm of mean diameter): it was removed after birth and necrosis was reported.

When comparing the two groups (cysts that showed spontaneous regression vs. cysts that underwent surgery), we found that the group of cysts that showed spontaneous regression had a significantly lower mean diameter, both prenatally (*p* value 0.039) and postnatally (*p* value < 0.001). The ultrasound features of some of the cysts analyzed are shown in Figure 2, Figure 3, Figure 4, Figure 5 and Figure 6.

## 4. Discussion

In recent years, several studies were published about fetal ovarian cyst outcomes and optimal prenatal management, but their postnatal treatment is still unclear; in particular, the indications for postnatal surgery are quite heterogeneous [18,28,29]. For asymptomatic and functional cysts, a conservative approach is usually preferred. However, surgery is indicated if a spontaneous cystic regression does not occur within 6 months, if dimensions exceed 5 cm or increase during follow-up, or if there is suspicion of adnexal torsion or gastroenteric or urinary tract compression. In case surgery is performed, care should be taken in preserving the ovarian parenchyma; however, in most cases, saving the functionality of the affected ovary is challenging. In cases of ovarian torsion, adnexal detorsion can be attempted; however, adnexectomy might be necessary. It has been suggested, however, that most torsions occur prenatally; therefore, the risk of postnatal torsion is low [30].

In the last decades, efforts have been made to define which ultrasound prenatal features may help anticipate complications and therefore orient towards surgery rather than conservative management [17,18,19,25,27].

Bascetto et al. [17] showed that a wide proportion of fetal ovarian cysts spontaneously disappear during pregnancy or postnatally. Simple unilocular anechoic cysts are those more likely to regress postnatally, while those with a complex aspect or those that change their ultrasound features are at increased risk of adnexal loss. The ultrasound characteristics that have the greatest impact on the need for surgery are cystic dimensions (greater or less than 40 mm) and cystic ultrasound appearance (simple vs. complex), as shown also by our cases. Furthermore, wider dimensions are correlated with a greater risk of complications, with a higher risk of ovarian torsion for cysts between 30 and 59 mm [19,23,25].

The main goal of prenatal and immediate postnatal evaluation is to establish which of those cysts are at high risk of torsion or which ovaries are already twisted, with an appropriate selection of those newborns who might benefit from surgery. Therefore, prenatal and then postnatal dimensions affect the risk of subsequent chance of surgery because of a higher risk of torsion that may promptly shift from conservative to surgical management, but this is not the only parameter that has to be considered. The ultrasound criteria that allow the diagnosis of adnexal torsion in adult women are well established [31], such as stromal edema, peripheral distribution of follicles, and whirlpool sign on Power Doppler. In the prenatal period, however, torsion was considered in the event of a change in the ultrasound characteristics or in case of hemorrhagic content of the cyst. This, however, as also shown in our study, is not an unequivocal sign of torsion but rather of intracystic hemorrhage. In fact, the cyst is not necessarily twisted and may indeed undergo spontaneous resolution [27].

Among the 19 simple anechoic cysts considered in our series, 8 were managed surgically (42.1%) because of their large dimensions or because they did not disappear during follow-up; in this group, only 5 presented with necrosis at histopathological examination (26%), and all were treated with laparoscopic cystectomy with ovarian preservation. However, all the hemorrhagic and low-level cysts that persisted after delivery and underwent surgery presented with necrosis at the histopathological examination.

Other elements that we found associated with an unfavorable outcome were cystic dimensions and ultrasound feature modifications during pregnancy. Those cysts showing a spontaneous resolution had a significantly smaller diameter than those undergoing surgical treatment, both prenatally and after birth. In addition, a surgical approach was chosen for all the cysts that had increased their dimensions or had changed their ultrasound appearance (from simple to complex cyst) during pregnancy, and in all of them histopathologic examination revealed necrosis. In one case, adnexal torsion occurred postnatally (the cyst involved had had a dimensional regression during pregnancy and again a progression during postnatal follow-up).

The main limits of our study are its retrospective nature and the scarce number of cases included, due to the rarity of fetal ovarian cysts and because of the monocentric setting of this study. In addition, we did not investigate how prenatal intervention and specifically intrauterine prenatal cyst aspiration might have an impact on neonatal outcome.

## 5. Conclusions

Our data adds valuable information about ultrasound diagnostic sensibility and its role in predicting the prognosis of fetuses with a prenatal diagnosis of ovarian cysts. Maybe a conservative approach can be proposed for anechoic cysts, even with larger dimensions, because the chance of ovariectomy is very low. Even in the presence of hemorrhagic content, there is the chance of spontaneous resolution, showing that this is a sign of prenatal intracystic bleeding but not always indicative of adnexal torsion.

Further studies are needed to improve our competence in the diagnosis of prenatal and postnatal ovarian torsion, as to these days in a significant number of cases the histopathologic evaluation does not show the presence of necrosis.

## Figures and Tables

**Table 1:** Outcomes of ovarian fetal cysts classified according to IOTA terminology.

IOTA Classification of Ovarian Cysts	Total	Spontaneous Regression	Postnatal Surgery	Presence of Necrosis at Histological Examination Among Those Cysts Removed
Anechoic	22 *	8/16 (50%)	8/16 (50%)	5/8
Low-level	2	0/2 (0%)	2/2 (100%)	2/2 (100%)
Ground glass	0	0	0	0
Hemorrhagic	5 **	1/4 (25%)	3/4 (75%)	3/3 (100%)
Mixed	2	1/2 (50%)	1/2 (50%)	1/1 (100%)
Others	0	0	0	0

* 3 cases were lost at follow-up, and 3 cases were not confirmed postnatally (postnatal diagnosis of multifollicular ovary). ** 1 case was not confirmed at histological examination.

## Data Availability

The data presented in this study are available on request from the corresponding author.
